# Some Queer Definitions in Hospital Diet

**Published:** 1911-09-30

**Authors:** Leonard D. Rea

**Affiliations:** Secretary and General Superintendent. Cardiff Infirmary.


### INSTITUTIONAL LETTER-BOX.

### SOME QUEER DEFINITIONS IN HOSPITAL DIET.

*To the Editor of* The Hospital.

Sir,—Mr. E. W. Morris' comments under the above heading in your issue of the 23rd inst. have given me a double interest, as they have provoked a reference to your account of Mr. Conrad Thies' interview with the *Sunday School Chronicle*, which has escaped my attention. I would venture to suggest that you should invite the secretaries of other hospitals, where the patients provide articles of food, to contribute their experience of the practical working of the system. I believe the practice obtains at many hospitals, including the Queen's Hospital, Birmingham, the Bristol Royal Infirmary, and the Wolverhampton and Staffordshire Hospital.

My impression, but for which I have no warrant, is that the regulation is but loosely observed, but I am confident that the underlying principle is, as Mr. Morris suggests, to encourage the patients to provide the ordinary requirements of their daily consumption, which the average patient in a modern hospital is quite well able to do. Tea, sugar, and butter would naturally suggest themselves as commodities that could be kept from day to day, where individual contributions of bread or milk would prove impracticable. I believe in some hospitals the patients are required to bring soap. Is it not the same idea—that this is an article of their normal daily use?—although in some instances the assumption may not be justified by the appearance of the patients.

I cannot follow Mr. Thies' deduction that the alcohol bill decreases in consequence of tea, sugar, and butter being supplied by a hospital. I think that within recent years, in practically every hospital, the alcohol bill has for very different reasons been reduced to a relatively negligible sum, although the economy is in one sense illusory, since so many substitutes for alcohol are now prescribed, as, for instance, the various preparations of malted beef wines.

I am sure that others, besides myself, would be interested to know how the system works in actual practice in a modern hospital of patients contributing their own tea, sugar, and butter, for I am convinced that, even if no great pecuniary advantage may accrue to an institution, it is worth a little trouble to give an opportunity to a patient to help himself where his circumstances enable him to do so.

It is really demoralising to give a man everything for nothing. It must add to his happiness and self-respect that he is enabled to provide something. We find at this hospital that the patients cheerfully contribute a substantial sum towards the appliances, etc., provided them by our excellent auxiliary—"The Cardiff Infirmary Samaritan Fund"—whilst the new system introduced this year of asking each in-patient to contribute 6d. per day towards his "food" (or maintenance) carries out in a manner more convenient and more profitable the same principle which requires the patients in certain other hospitals to contribute in kind some of the articles of daily consumption.

Faithfully yours,
Leonard D. Rea,
Secretary and General Superintendent.

Cardiff Infirmary.
September 25.

